# Native T1 mapping for differentiating the histopathologic type, grade, and stage of rectal adenocarcinoma: a pilot study

**DOI:** 10.1186/s40644-022-00461-7

**Published:** 2022-06-17

**Authors:** Juan Li, Xuemei Gao, Marcel Dominik Nickel, Jingliang Cheng, Jinxia Zhu

**Affiliations:** 1 Department of MRI, the First Affiliated Hospital of Zhengzhou University, No.1, Jianshe Dong Road, Zhengzhou, 450052 China; 2 Siemens Healthcare GmbH, 91052 Erlangen, Germany; 3 MR Collaboration, Siemens Healthcare Ltd, Beijing, 100000 China

**Keywords:** T1 mapping; Rectal neoplasms, Adenocarcinoma, Magnetic resonance imaging

## Abstract

**Background:**

Previous studies have indicated that T1 relaxation time could be utilized for the analysis of tissue characteristics. T1 mapping technology has been gradually used on research of body tumor. In this study, the application of native T1 relaxation time for differentiating the histopathologic type, grade, and stage of rectal adenocarcinoma was investigated.

**Methods:**

One hundred and twenty patients with pathologically confirmed rectal adenocarcinoma were retrospectively evaluated. All patients underwent high-resolution anatomical magnetic resonance imaging (MRI), diffusion-weighted imaging (DWI), and T1 mapping sequences. Parameters of T1 relaxation time and apparent diffusion coefficient (ADC) were measured between the different groups. The diagnostic power was evaluated though the receiver operating characteristic (ROC) curve.

**Results:**

The T1 and ADC values varied significantly between rectal mucinous adenocarcinoma (MC) and non-mucinous rectal adenocarcinoma (AC) ([1986.1 ± 163.3 ms] vs. [1562.3 ± 244.2 ms] and [1.38 ± 0.23 × 10^−3^mm^2^/s] vs. [1.03 ± 0.15 × 10^−3^mm^2^/s], respectively; *P* < 0.001). In the AC group, T1 relaxation time were significantly different between the low- and high-grade adenocarcinoma cases ([1508.7 ± 188.6 ms] vs. [1806.5 ± 317.5 ms], *P* < 0.001), while no differences were apparent in the ADC values ([1.03 ± 0.14 × 10^−3^mm^2^/s] vs. [1.04 ± 0.18 × 10^−3^mm^2^/s], *P* > 0.05). No significant differences in T1 and ADC values were identified between the different T and N stage groups for both MC and AC (all *P* > 0.05).

**Conclusions:**

Native T1 relaxation time can be used to discriminate MC from AC. The T1 relaxation time was helpful for differentiating the low- and high-grade of AC.

## Background

Colorectal cancer is a common malignancy of the digestive tract, for which 30–35% of the cases occur in the rectum, and 90% are classified as adenocarcinomas [1, 2]. Studies have shown that the incidence and mortality of colorectal cancer have been increasing over time [3]. Currently, neoadjuvant chemoradiotherapy and surgical resection are the most effective treatments for rectal cancer. Many factors are associated with the overall therapeutic efficacy of rectal cancer, including the histologic grade, tumor type, and pathologic T and N staging [4–6]. Rectal mucinous adenocarcinoma (MC) is a common subtype of rectal adenocarcinoma pathologically characterized by tumor cell hypersecretion, with the mucus content in the tumor parenchyma exceeding 50%. It has a poor prognosis and is insensitive to neoadjuvant chemoradiotherapy [6, 7]. Accurate determination of the pathologic type and degree of tumor differentiation is essential for achieving individualized treatment plans for patients with rectal cancer.

Magnetic resonance imaging (MRI) is the first choice for preoperative diagnosis and staging of rectal cancer [8]. Conventional high-resolution MRI not only distinguishes the various layers of the rectal wall clearly, but also displays peripheral anatomical structures [9, 10]. Diffusion-weighted imaging (DWI) reflects changes in tissue microenvironments under physiologic or pathologic conditions by measuring the diffusion of water molecules in tissues. It had been previously applied to tumor TN staging, grading, and prognosis of rectal cancer. However, the results were lack of consistency [11]. Zhu et al. found that the ADC values of low-grade adenocarcinoma were higher than those of high-grade adenocarcinoma, but the difference was not statistically significant [12].

T1 mapping is a technique that measures the longitudinal relaxation time (T1) for quantitative analyses of biological tissue characteristics [13, 14]. In T1 mapping, the T1 relaxation time of tissues provide a quantitative analysis of the changes in the internal composition of tissues. Previously, T1 mapping was primarily used to assess myocardial diseases [15, 16]. In addition, the use of this technology has been gaining ground in liver and kidney function evaluations and liver fibrosis [17–20]. In recent years, T1 mapping has been applied to cancer research as it exhibits an excellent ability of tumor differentiation, grading, and recurrence. For instance, Qin et al*.* suggested that T1 relaxation time would be beneficial for predicting the grade and recurrence of hepatocellular carcinoma [21]. Adams et al*.* found that T1 relaxation time can be used to distinguish high- and low-grade renal cell carcinoma [22]. However, few studies have investigated T1 relaxation time in rectal cancer. Therefore, in this study, we investigated using T1 relaxation time to identify, grade, and stage rectal adenocarcinoma using DWI as a control, with the goal of providing a reference value for the clinical evaluation of rectal adenocarcinoma.

## Methods

### Patients

**Fig. 1:**
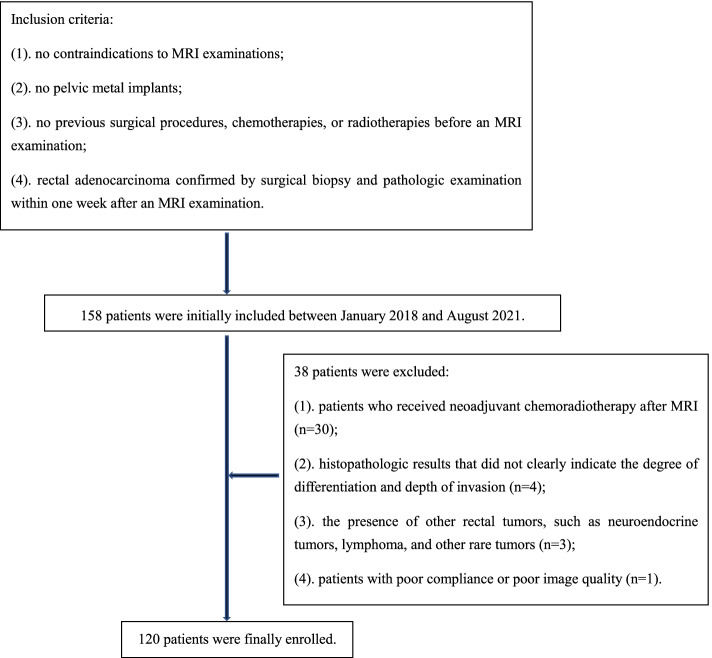
Flowchart of patient selection

One hundred and fifty-eight patients with pathologically confirmed rectal cancer were collected from our hospital database between January 2018 and August 2021. The inclusion criteria and exclusion criteria were represented in Fig. 1. Finally, we included 120 patients in this study.

### MR examination

All MRI examinations were performed on a 3 T system (MAGNETOM Prisma, Siemens Healthcare, Erlangen, Germany) with an 18-channel phased-array body coil and the lower part of a 32-channel spine coil. Patients were instructed to empty their rectum before examination. For patients without intestinal obstruction, prostate hyperplasia, glaucoma, or other contraindications to anisodamine, intramuscular injection of 20 mg raceanisodamine hydrochloride injection (Suicheng Pharmaceutical Co, Ltd. Zhengzhou, China) was given 5–10 min before examination to suppress intestinal movement artifacts and obtain a satisfactory image. Patients were placed in a supine position with the oblique axis imaging positioned perpendicular to the long axis of the lesion. The scanning sequences included an oblique axial T_2_W turbo spin-echo (TSE) sequence, axial DWI sequence, and a prototypic T1 mapping sequence. All sequences and corresponding parameters are listed in Table 1. DWI was performed using a single-shot echo plane imaging (ss-EPI) sequence with two b-values (50 and 800 s/mm^2^). ADC map was generated inline after data acquisition using monoexponential model. T1 map was acquired using a prototypic inversion recovery snapshot FLASH sequence (Siemens Healthcare, Erlangen, Germany). After an initial 180° inversion pulse, 16 FLASH acquisitions were acquired at different time-points on the relaxation recovery curve [23]. T1 parametric map was generated inline after data acquisition.

**Table 1:** Magnetic resonance imaging acquisition parameters

	T_2_WI TSE	DWI	T1 mapping
TR (ms)	3200	4050	3
TE (ms)	101	48	1.32
Field of view (mm^2^)	200 × 200	300 × 225	380 × 308
Slice thickness (mm)	3	3	4
No. of slices	20	20	20
Acquisition matrix	320 × 240	168 × 126	192 × 125
Reconstructed voxel size (mm^3^)	0.3 × 0.3 × 3.0	1.8 × 1.8 × 3.0	1.0 × 1.0 × 4.0
Parallel imaging factor	2	2	2
b-values (s/mm^2^)	NA	50/800	NA
Flip angle (°)	160	180	8
Bandwidth (Hz/pixel)	200	992	1530
Averages	3	1 (b = 50 s/mm^2^) & 2 (b = 800 s/mm^2^)	1
Acquisition time	3min20s	3min28s	1min32s

*T*_*2*_*WI* T_2_-weighted imaging, *TSE* Turbo spin-echo, *DWI* Diffusion-weighted imaging, *TR* Repetition time, *TE* Echo time, *NA* Not applicable

### Imaging analysis

All data were analyzed on a commercially available post-processing workstation (Syngo.Via, Siemens Healthcare, Erlangen, Germany). Measurements based on high-resolution T_2_W images were performed in a double-blinded manner by two attending physicians experienced in MRI diagnoses. Regions of interest (ROIs) on T1 map images were manually selected for observation, measurement, and analysis. The criteria were as follows: the ROI was drawn at the level of the maximum extent of the tumor and the nearest levels above and below it, the average values were taken, avoiding the necrotic and hemorrhagic regions of the capsule as much as possible. The same ROI was translated to the ADC image for measuring ADC values. To ensure consistency and reduce measurement errors caused by selection bias in ROI positioning, all data were measured in triplicate and parameters were averaged.

### Pathologic grade and stage

According to the World Health Organization (WHO) grading criteria, non-mucinous adenocarcinoma (AC) is classified as grade 1 (G1, well-differentiated, > 95% gland forming), grade 2 (G2, moderately differentiated, 50–95% gland forming), or grade 3 (G3, poorly differentiated, 0–49% gland forming). G1 and G2 tumors are classified as low-grade tumors, and G3 tumors are classified as high-grade tumors. T staging is classified as early- and late-stage based on the depth of tumor invasion per the staging criteria of the American Joint Committee on Cancer (AJCC). Early-stage cancer was defined as disease confined to the muscularis propria, including stages pT1 and pT2, and late-stage cancer was defined as disease extending beyond the muscularis propria, including stages pT3 and pT4. Lymph node staging was performed based on the results of postoperative pathology. Lack of regional lymph node metastases included stage pN0, and regional lymph node metastasis included stages pN1-2.

### Statistical analysis

SPSS 22.0 software (IBM, Armonk, NY) and MedCalc v. 20.0 (MedCalc Software, Ostend, Belgium) were used for the statistical analysis. Measurement data were represented as mean ± standard deviation. The t-test for independent samples (normally distributed and homoscedastic data) and the Mann–Whitney U test (skewed or heteroscedastic data) were used to compare each parameter between the pathologic types, WHO grades (G1-2 vs G3), pT stages (early vs late), and pN stages (N0 vs N1-2). Differences with a *p* < 0.05 were considered statistically significant. The receiver operating characteristic (ROC) curve of each parameter was plotted, the area under the ROC curve (AUC) was calculated; the best diagnostic threshold for each parameter was determined based on the maximum Youden’s index (Youden’s index = sensitivity + specificity—1), and the diagnostic power of T1 and ADC values in identifying MC and AC, and the grade and stage of rectal adenocarcinoma were evaluated.

## Results

### Rectal adenocarcinoma grading and staging

**Fig. 2:**
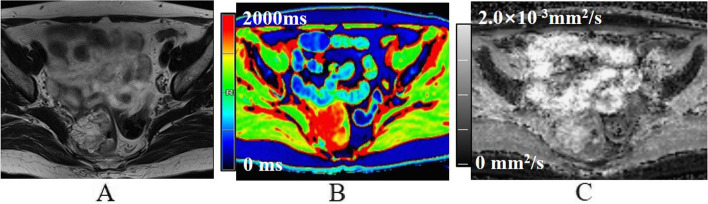
A 60-year-old female with mucinous adenocarcinoma. **A** Oblique axial T_2_-weighted image shows a mass with high-intensity signals in the rectum. **B** The mass is red on the T1 mapping. The T1 relaxation time was 1840.0 ms. **C** The mass shows high-intensity signals on the ADC map. The ADC value was 1.92 × 10^−3^mm^2^/s

**Fig. 3:**
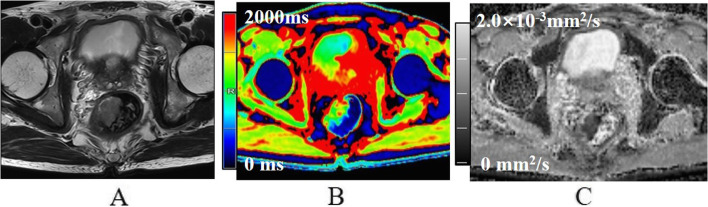
A 64-year-old male with low-grade non-mucinous adenocarcinoma. **A** Oblique axial T_2_-weighted image shows a mass with a slight high-intensity signal in the rectum. **B** The mass is primarily green on T1 mapping. T1 relaxation time was 1362.6 ms. **C** The mass showed low intensity on the ADC map. The ADC value was 0.80 × 10^−3^mm^2^/s

**Fig. 4:**
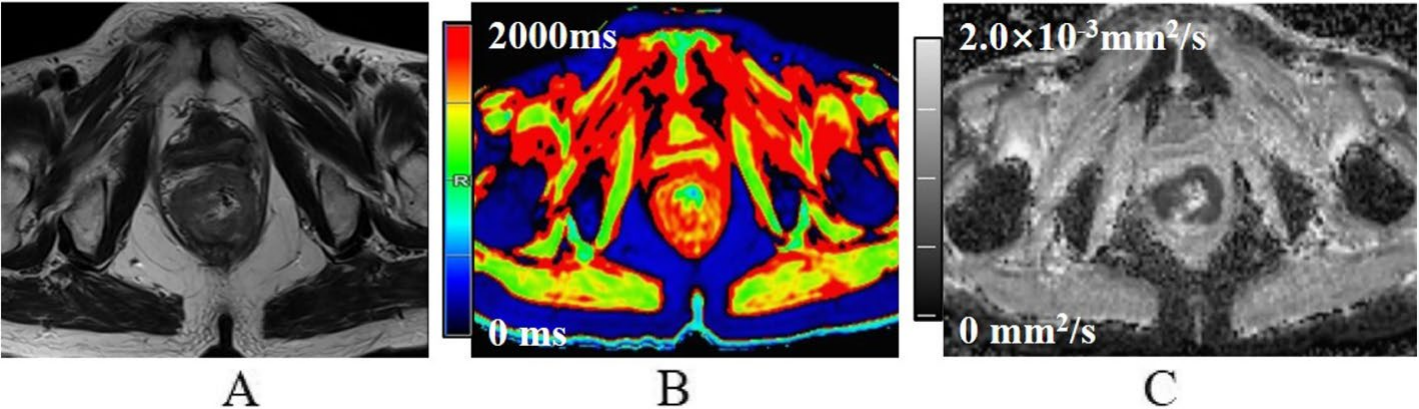
A 71-year-old female with high-grade non-mucinous adenocarcinoma. **A** Oblique axial T_2_-weighted image shows a mass with a slight high-intensity signal in the rectum. **B** The mass showed mostly red on T1 mapping. T1 relaxation time was 1641.3 ms. **C** The mass showed low intensity on the ADC map. The ADC value was 0.85 × 10^−3^mm^2^/s

Of 120 rectal adenocarcinomas, 20 were classified as MC, and 100 were classified as AC based on the results of postoperative pathology. In the AC group, 82 G2 tumors were classified as low-grade and 18 G3 tumors as high-grade adenocarcinomas; no tumors were classified as G1 (Figs. 2, 3 and 4). The clinical features, histopathologic types, grades, and stages are summarized in Table 2.

**Table 2:** Clinical and pathologic characteristics of the study patients

Characteristics	Number of patients
Gender	
Male	61
Female	59
Age	
Mean age, years	58 ± 12
Age range, years	28–83
Pathology	
MC	20
T stage	
pT1-2	5
pT3-4	15
N stage	
pN0	6
pN1-2	14
AC	100
WHO grade	
Low-grade	82
High-grade	18
T stage	
pT1-2	37
pT3-4	63
N stage	
pN0	60
pN1-2	40

*MC* Mucinous adenocarcinoma, *AC* Non-mucinous adenocarcinoma, *WHO* World Health Organization

### Comparison of T1 and ADC values in the different rectal adenocarcinoma groups

The T1 and ADC values of MC were both significantly higher than those of AC (*P* < 0.001) (Table 3). The T1 relaxation time of the low-grade AC groups were significantly lower than that of the high-grade AC group (*P* < 0.001), and the ADC values were not statistically significant in the different grade AC group (*P* > 0.05). The T1 relaxation time and the ADC values of different T and N stages were not statistically significant (all *P* > 0.05) (Table 4, 5).

**Table 3:** A comparison of T1 and ADC values in mucinous adenocarcinoma (MC) and non-mucinous adenocarcinoma (AC)

Pathologic type	T1 relaxation time(ms)	ADC value(× 10^−3^mm^2^/s)
MC(*n* = 20)	1986.1 ± 163.3	1.38 ± 0.23
AC(*n* = 100)	1562.3 ± 244.2	1.03 ± 0.15
*P* value	0.000	0.000

*ADC* Apparent diffusion coefficient

**Table 4:** A comparison of the T1 and ADC values ​​in differently staged mucinous adenocarcinoma (MC) groups

Groups	T1 relaxation time(ms)	ADC value(× 10^−3^mm^2^/s)
T stage		
pT1-2(*n* = 5)	1934.8 ± 140.4	1.36 ± 0.14
pT3-4(*n* = 15)	2003.1 ± 171.2	1.39 ± 0.26
*P* value	0.513	0.753
N stage		
pN0(*n* = 6)	1981.7 ± 184.4	1.34 ± 0.32
pN1-2(*n* = 14)	1987.9 ± 160.9	1.41 ± 0.20
*P* value	0.805	0.553

**Table 5:** A comparison of the T1 and ADC values ​​in differently staged non-mucinous adenocarcinoma (AC) groups

Groups	T1 relaxation time(ms)	ADC value(× 10^−3^mm^2^/s)
WHO grade		
Low-grade(*n* = 82)	1508.7 ± 188.6	1.03 ± 0.14
High-grade(*n* = 18)	1806.5 ± 317.5	1.04 ± 0.18
*P* value	0.000	0.914
T stage		
pT1-2(*n* = 37)	1529.9 ± 185.5	1.02 ± 0.15
pT3-4(*n* = 63)	1581.3 ± 272.5	1.04 ± 0.15
*P* value	0.451	0.595
N stage		
pN0(*n* = 60)	1540.3 ± 242.3	1.03 ± 0.14
pN1-2(*n* = 40)	1595.3 ± 246.4	1.05 ± 0.15
*P* value	0.263	0.547

*WHO* World Health Organization

### Diagnostic performance of T1 and ADC values for distinguishing MC from AC and low-grade AC from high-grade AC

**Fig. 5:**
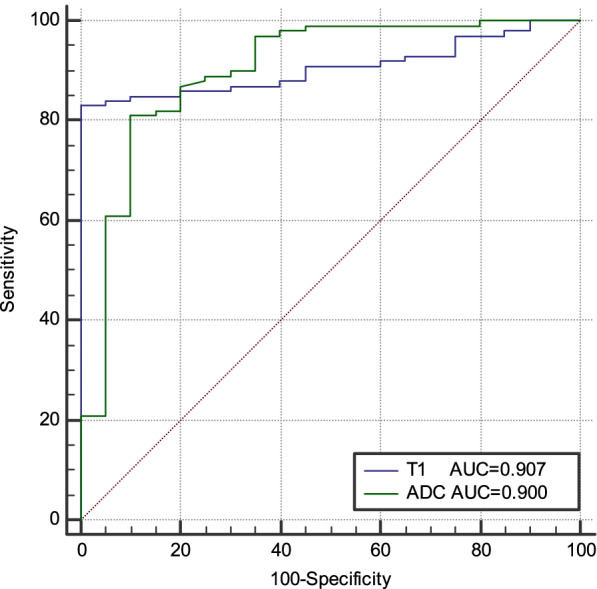
ROC curves of the T1 and ADC values distinguishing mucinous and non-mucinous adenocarcinoma

**Fig. 6:**
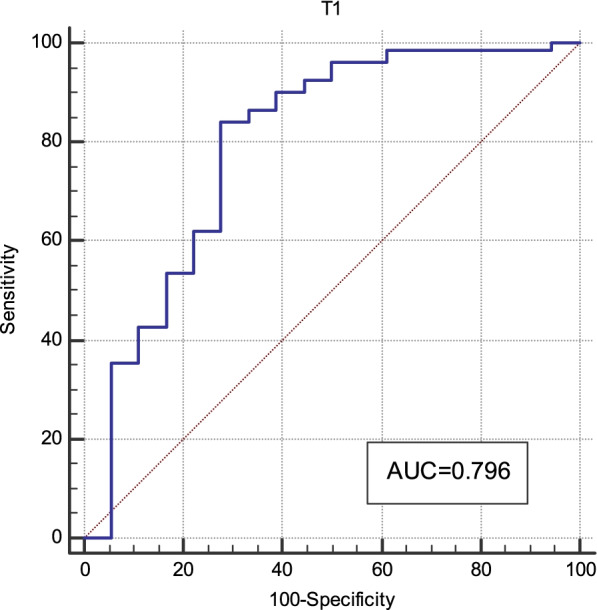
The ROC curve of T1 relaxation time distinguishing low- and high-grade non-mucinous adenocarcinoma

The AUC for distinguishing MC from AC using the T1 relaxation time was 0.907, with 1782.3 ms as the optimal diagnostic threshold; the diagnostic sensitivity and specificity were 83.0% and 100%, respectively. The AUC for distinguishing MC from AC using the ADC value was 0.900, with 1.17 × 10^–3^ mm^2^/s as the optimal diagnostic threshold; the diagnostic sensitivity and specificity were 81.0% and 90.0% (Fig. 5), respectively. The AUC for distinguishing low- from high-grade AC using the T1 relaxation time was 0.796, with 1626.0 ms as the optimal diagnostic threshold; the diagnostic sensitivity and specificity were 84.2% and 72.2%, respectively (Fig. 6). The diagnostic performance and optimal diagnostic threshold of T1 and ADC values are shown in Table 6.

**Table 6:** Diagnostic performance of the T1 and ADC values

Parameter	P value	AUC (95% CI)	Threshold	Sensitivity (%)	Specificity (%)
T1_p_-_MC/AC_	0.000	0.907(0.840–0.952)	1782.3 ms	83.0	100
ADC_p_-_MC/AC_	0.000	0.900(0.832–0.947)	1.17 × 10^−3^mm^2^/s	81.0	90.0
T1_p_-_l/h_	0.000	0.796(0.704–0.870)	1626.0 ms	84.2	72.2

*AUC* Area under the curve, *CI* Confidence interval, *T1*_*p-MC/AC*_ T1 relaxation time of mucinous (MC) and non-mucinous (AC) adenocarcinoma, *ADC*_*p-MC/AC*_ ADC values of MC and AC, *T1*_*p-l/h*_ T1 relaxation time of low- and high-grade AC

## Discussion

In our study, we performed a preliminary investigation of the feasibility of T1 relaxation time for the preoperative evaluation of histopathologic type, grade, and stage of rectal adenocarcinoma, combined with T_2_W and DWI sequences. T1 and ADC values could distinguish MC from AC. In addition, our results showed that T1 relaxation time had greater diagnostic ability in differentiating high- from low-grade AC than ADC. Native T1 relaxation time could represent a non-invasive biomarker for evaluating rectal adenocarcinoma.

In the present study, the T1 and ADC values of MC were significantly higher than those of AC. The AUC for distinguishing MC from AC using T1 and ADC values were 0.907 and 0.900, respectively, indicating high sensitivity and specificity. Tissue T1 relaxation time is associated with a variety of biological factors, such as macromolecule concentration, water binding status, and tissue water content. MC is characterized by tumor cell hypersecretion, with more than 50% of mucus content in the tumor parenchyma [5], which may have contributed to a high T1 relaxation time. ADC values are associated with the density, atypia, and size of intercellular tumor substances. Mucinous adenocarcinoma cells float on a layer of mucus in a relatively loose arrangement, and the movement of water molecules is relatively small with relatively limited restrictions on the diffusion; therefore, ADC values are higher relative to cells of AC [24, 25]. This is consistent with the results of the present study, and thus T1 relaxation time and ADC values could be used to discriminate MC from AC preoperatively.

The histologic grade is an independent prognostic factor in patients with rectal adenocarcinoma. Studies have shown that the T1 relaxation time of malignant breast tumors are higher than those of benign tumors [26]. The T1 relaxation time of hepatocellular carcinoma increase with decreasing degrees of differentiation [21]. Native T1 relaxation time can be used to distinguish between high- and low-grade renal cell carcinoma, as T1 relaxation time increase with increasing pathologic grade [22, 27]. In this study, the T1 relaxation time of low-grade adenocarcinoma were significantly lower than those of high-grade adenocarcinoma. The AUC for distinguishing low-grade from high-grade adenocarcinoma was 0.796, and the sensitivity and specificity were 84.2% and 72.2%, respectively. The T1 relaxation time might reflect the alteration of tissue composition; the increased T1 relaxation time in high-grade rectal AC could be ascribed to higher water content, lower levels of soluble protein, and higher cell proliferation in high-grade vs low-grade tumors [28]. This indicates that T1 relaxation time can accurately identify the pathologic grade of rectal cancer and be used to determine the degree of tumor cell malignancy. In the present study, the difference of ADC values in low-grade and high-grade adenocarcinoma was not statistically significant, which was in accordance with previous research [12]. These studies indicated that ADC values cannot be used to distinguish different degrees of differentiation.

The selection of treatment options benefits greatly from accurate preoperative T staging. In the present study, postoperative pathologic T staging was used to retrospectively analyze the correlation between T1 and ADC values and T stages. The T1 relaxation time and the ADC values of the different T stages were not statistically significant. Previous studies have also shown that ADC values cannot be used at the different T stages of rectal cancer [12, 29], which could be because the differences in tumor microenvironments at the different T stages are insufficient to cause significant changes in the T1 and ADC values.

Lymph node metastasis is an important factor in the formulation of treatment plans and prognostic predictions for patients with rectal cancer. Lymph node size, shape, border, and signal are commonly used as criteria to design therapeutic strategies [30, 31]. Besides, Chen et al. concluded that the presence of calcifications within a regional lymph node indicates metastasis in rectal cancer [32], but the accuracy of these criteria is lacking. Ge et al*.* found that T_2_ and ADC values could distinguish metastatic and non-metastatic lymph nodes [33]. However, some other studies have found that ADC values should not be used to determine lymph node status [29, 34]. In the present study, the T1 relaxation time and the ADC values of the different N stages were not statistically significant. This finding could be caused by not assessing lymph nodes suspected of metastasis on MRI separately. Therefore, the significance of T1 relaxation time and ADC values in predicting lymph node metastasis requires further study.

The present study has some limitations. First, no case of well-differentiated rectal adenocarcinoma was included, and its effect on grading and staging requires the inclusion of additional relevant cases and continued research. Thus, further studies with a larger population are needed. Second, the choice of different ROIs might also lead to differences in study results due to tumor heterogeneity. The ROI used in the present study was manually drawn at the level of the maximum extent of the tumor and the nearest levels above and below it. In the future, the entire tumor can be included to determine whether the tumor volume is more meaningful for tumor grading and staging analyses. Finally, this study did not analyze the correlation between T1 relaxation time, immunohistochemical indicators, and gene expression, and did not include patient prognoses. In future studies, collection of a complete data set for more in-depth research is needed.

## Conclusions

In summary, Native T1 relaxation time can distinguish MC from AC. The T1 relaxation time can help determine the low- and high-grade AC, better than the ADC value. However, whether T1 or ADC values can distinguish T stage and N stage remains debatable.

## Data Availability

All data generated or analyzed during this study are included in this published article. The datasets are available from the corresponding author on reasonable request.

## Abbreviations

AC: Non-mucinous rectal adenocarcinoma
ADC: Apparent diffusion coefficient
AUC: Area under the curve
AJCC: American Joint Committee on Cancer
CI: Confidence interval
DWI: Diffusion-weighted imaging
MC: Mucinous adenocarcinoma
MRI: Magnetic resonance imaging
ROC: Receiver operating characteristic
ROI: Region of interest;
ss-EPI: Single-shot echo plane imaging
T_2_W: T_2_-weighted
TSE: Turbo spin-echo
TA: Acquisition time
TE: Echo time
TR: Repetition time
WHO: World Health Organization
